# USP15 and USP4 facilitate lung cancer cell proliferation by regulating the alternative splicing of SRSF1

**DOI:** 10.1038/s41420-022-00820-0

**Published:** 2022-01-13

**Authors:** Tanuza Das, Eun-Young Lee, Hye Jin You, Eunice EunKyeong Kim, Eun Joo Song

**Affiliations:** 1 Biomedical Research Institute, Korea Institute of Science and Technology, Hwarangno 14-gil 5, Seongbuk-gu, Seoul, 02792 Republic of Korea; 2 Division of Translational Science, Research Institute, National Cancer Center, 323 Ilsan-ro, Ilsadong-gu, Goyang, Gyeonggi 10408 Republic of Korea; 3 Graduate School of Pharmaceutical Sciences, College of Pharmacy, Ewha Womans University, Seoul, 03760 Republic of Korea

**Keywords:** Oncogenes, Ubiquitylation

## Abstract

The deubiquitinating enzyme USP15 is implicated in several human cancers by regulating different cellular processes, including splicing regulation. However, the underlying molecular mechanisms of its functional relevance and the successive roles in enhanced tumorigenesis remain ambiguous. Here, we found that USP15 and its close paralog USP4 are overexpressed and facilitate lung cancer cell proliferation by regulating the alternative splicing of SRSF1. Depletion of USP15 and USP4 impair SRSF1 splicing characterized by the replacement of exon 4 with non-coding intron sequences retained at its C-terminus, resulting in an alternative isoform SRSF1-3. We observed an increased endogenous expression of SRSF1 in lung cancer cells as well, and its overexpression significantly enhanced cancer cell phenotype and rescued the depletion effect of USP15 and USP4. However, the alternatively spliced isoform SRSF1-3 was deficient in such aspects for its premature degradation through nonsense-mediated mRNA decay. The increased USP15 expression contributes to the lung adenocarcinoma (LUAD) development and shows significantly lower disease-specific survival of patients with USP15 alteration. In short, we identified USP15 and USP4 as key regulators of SRSF1 alternative splicing with altered functions, which may represent the novel prognostic biomarker as well as a potential target for LUAD.

## Introduction

Ubiquitin-specific protease 15 (USP15), a deubiquitinating enzyme (DUB), is ubiquitously expressed in various tissues and organs. USP15 is involved in numerous cellular processes such as spliceosomal regulation, T cell response, neuroinflammation, antiviral immune response, regulation of COP9 signalosome, cell cycle, gene expression, DNA repair, maintenance of genome integrity, signal transduction, and more [1–9]. USP15 is implicated in different cancer-associated mechanisms, e.g., regulating TGF-β–dependent oncogenesis in glioblastoma [6], as well as promoting breast and ovarian cancer progression by stabilizing ERα and p53 mutant, suggesting it as a potential cancer therapeutic target [10–12]. USP4, the closest paralogue of USP15, also influences several tumorigenic properties and plays critical roles in different physiological and pathological processes including tumor initiation and progression [13]. Therefore, both USP15 and USP4 may be considered important prognostic biomarkers in human cancers. Earlier, we found that USP15 regulates dynamic protein–protein interactions of the spliceosomal machinery and the depletion of USP15 interferes with proper mRNA splicing of the cell cycle regulatory genes Bub1 and α-tubulin [1]. While defects in RNA splicing are involved in various diseases including cancer [14, 15], it was not fully investigated the splicing of which genes are regulated by USP15 and whether the regulated splicing affects cancer progression.

In humans, ~95% of all genes undergo alternative splicing resulting in multiple protein isoforms which may have different biological functions [16]. Splicing defects of many vital genes are associated with cancer [17–20], and cancer-associated alternative splicing has emerged as a promising field in basic and translational oncology [21]. Lung cancer is one of the most common [22], although the underlying mechanism behind the aberrant alternative splicing in lung cancer progression is still poorly understood. It has been proposed that the aberrant alternative splicing in several cancers might be the result of abnormal activity or expression of splicing regulatory proteins [23]. In particular, serine/arginine-rich splicing factor 1 (SRSF1), also known as pre-mRNA-splicing factor ASF1/SF2 is the archetype member of the SR family proteins [24–26] that are involved in several aspects of mRNA metabolic pathways like mRNA splicing, stability, nuclear export, translation, and nonsense-mediated mRNA decay (NMD) [27]. Besides regulating the AS of other target genes, SRSF1 itself undergoes alternative splicing resulting in additional isoforms. The major isoform encodes full-length of functional protein, whereas the others are either retained in the nucleus, encoded proteins with different functions, or degraded by NMD [28]. The full-length SRSF1 consists of 248 amino acids accounting for all of its known functions. Instead, the alternatively spliced isoforms are generated by skipping exons and/or retaining intron segments with the incorporation of the premature stop codon at the C-terminus [29]. SRSF1 is a proto-oncogene, emphasizing its important role in tumorigenesis [30–32]. Abnormal expression of SRSF1 is reported in several malignancies [33]. Nevertheless, the role of SRSF1 splice variants and their underlying regulatory factors or mechanisms are not yet fully understood.

In this study, we found that USP15 and USP4 regulate alternative splicing of SRSF1 resulting in the isoform-specific functions in lung cancer cell proliferation and migration. Additionally, the alteration frequency of USP15 in TCGA-LUAD data showed an increased USP15 expression that contributes to lung adenocarcinoma (LUAD) development. Furthermore, patients with USP15 alteration showed significantly lower disease-specific survival. Collectively, our current study demonstrates USP15 and USP4 mediated lung cancer cell proliferation and migration by increasing the expression of functional full-length SRSF1 protein.

## Results

### USP15 and USP4 promote cell proliferation and invasion in lung cancer cell lines

**Fig. 1:**
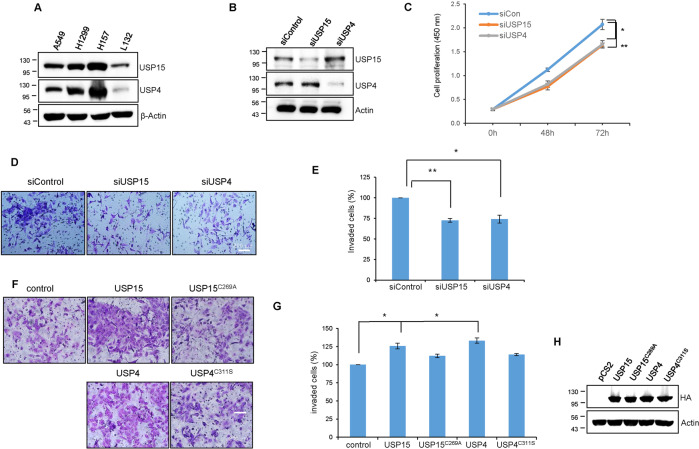
USP15 and USP4 promote cell proliferation and invasion in lung cancer cell line. **A** The endogenous expression of USP15 and USP4 in the lung cancer cells H157, H1299, and A549 were compared with the normal lung epithelial cell L132. **B** Knockdown of USP15 and USP4 with targeted siRNAs was confirmed in H157 cells. **C** H157 cells were transfected with USP15 and USP4 siRNAs and cell proliferation was measured at the indicated time point. **D** H157 cells were transfected with USP15 and USP4 siRNAs, harvested after 48 h of transfection, and reseeded the cells on a transwell containing the pre-gelled matrix. After 24 h of incubation, invaded cells on the lower chamber of the transwell were fixed, visualized with Giemsa stain, and imaged using a light microscope. **E** The percentage of invaded cells from (**D**) was counted and data from three independent experiments are shown in the statistical graph (*P < 0.05 and **P < 0.01, two-tailed student’s t-test). **F** The cell invasion was performed in a similar way after overexpression of USP15 and USP4 WT along with their corresponding active site mutants and data from three independent experiments are shown (**G**) (*P < 0.05 and **P < 0.01, two-tailed student’s t-test). **H** Overexpression of USP15 and USP4 WT, as well as the corresponding mutants were confirmed by western blot.

First, we examined the endogenous expression level in different lung cancer cells and found that both USP15 and USP4 overexpresses in the NSCLC cell lines; A549, H157, and H1299 compared to the normal lung epithelial L132 cell (Fig. 1A). Then, to examine the oncogenic roles, USP15 and USP4 were depleted using three different siRNA sequences. Both USP15 and USP4 were efficiently depleted with all the three tested siRNAs (Supplementary Fig. 1A, B, respectively), while siUSP15_1 and siUSP4_1 were used for the subsequent experiments (Fig. 1B). Depletion of USP15 and USP4 decreased cell proliferation in both H157 (Fig. 1C and Supplementary Fig. 1C, D) and A549 (Supplementary Fig. 1E). Also, the migration and invasion of USP15 and USP4 knockdown cells decreased significantly compared to the control (Fig. 1D, E, and Supplementary Fig. 1F). In contrast, ectopically overexpressed wild-type USP15 and USP4 promoted cell invasion, but the corresponding active site mutants, USP15^C269A^ and USP4^C311S^ lack the invasive ability in H157 cells (Fig. 1F–H) and A549 cells (Supplementary Fig. 1G, H). Collectively these data suggest that both USP15 and USP4 genes are overexpressed and facilitate lung cancer cell progression.

### Knockdown of USP15 and USP4 induces global changes in alternative splicing pattern

**Fig. 2:**
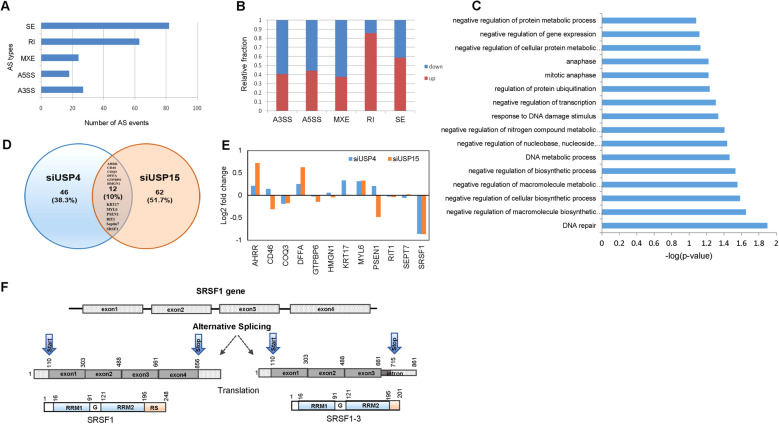
Global changes in alternative splicing pattern by USP15 knockdown. **A** The percentage of alternative splicing events upon USP15 knockdown among the five major types of alternative splicing (AS) event namely skipped exons (SE), alternative 5′ splice sites (A5SS), alternative 3′ splice sites (A3SS), retained introns (RI), and mutually exclusive exon usage (MXE) were displayed in the bar graph. **B** The relative fractions of genes upregulated or downregulated by the different alternative splicing events upon USP15 depletion are presented. **C** The alternatively spliced genes upon USP15 knockdown involved in diverse cellular pathways are classified and represented as distinct functional groups. **D** Venn diagram showing the common genes found to be regulated by both USP15 and USP4 knockdown. Twelve genes accounting for 10% of total genes whose intron retention was significantly regulated by knockdown of both USP15 and USP4 are listed. **E** A comparison of log_2_ fold change of the identified common genes expression level is shown as the bar graph. **F** The gene sequence and alternatively spliced isoforms of SRSF1 are schematically presented.

Both USP15 and USP4 regulate the activity and composition of the spliceosome and facilitate ubiquitin-dependent RNA splicing of the genes required for proper chromosome segregation [1, 34]. As aberrant alternative splicing is a critical cause of cancer development [18], we sought to find the genes regulated by USP15 and USP4, which may, in turn, affect the cancer progression phenotype. First, we generated H157 cell lines with stable knockdown of USP15 or USP4 (Clones #1 in Supplementary Fig. 2A, B), and subjected them to RNA sequencing for identifying the genes whose alternative splicing are regulated by USP15 and USP4. Analyses using MISO identified alternatively spliced gene patterns at the exon level. Upon USP15 and USP4 depletion, the five major types of alternative splicing, skipped exons (SE), alternative 5′ splice sites (A5SS), alternative 3′ splice sites (A3SS), retained introns (RI), and mutually exclusive exon usage (MXE), are shown in Fig. 2A and Supplementary Fig. 2C, respectively. SE and RI were the predominant alternative splicing events. The relative fraction of up or down-regulated genes upon USP15 and USP4 depletion are shown in Fig. 2B and Supplementary Fig. 2D, respectively. Most of the alternative splicing events were downregulated, while the RI event was upregulated by USP15 and USP4, consistent with our previous finding that the depletion of USP15 and USP4 results in the intron retention of genes such as Bub1 and α-tubulin [1]. Gene ontology analysis (Fig. 2C and Supplementary Fig. 2E) showed that alternatively spliced genes by USP15 and USP4 knockdown were involved in diverse cellular functions such as regulation of transcription, DNA repair, cell cycle, and so on.

Afterward, we focused on the group of RI genes regulated by the two DUBs, based on the RNA sequencing data and our previous findings [1]. We first selected 74 genes from USP15 and 58 genes from the USP4 knockdown group with the Bayes factor >100, representing the strong evidence correlation with the two DUBs. Twelve genes were common (Fig. 2D), and only SRSF1 showed analogous but substantial effects upon USP15 and USP4 depletion (Fig. 2E). Considering both DUBs are involved in the same cellular pathway [1] and the imperative role of SRSF1 in lung cancer progression [33], we focused on SRSF1 hereafter. The sequencing analysis revealed the skipping of exon 4 by introducing a premature stop codon in the retained intron, which is the same as isoform SRSF1-3. SRSF1 translates to a protein composed of 248 amino acids while alternatively spliced isoform SRSF1-3 consists of 201 amino acids, where the SRSF1 C-terminal exon 4 is deleted and incorporated 17 amino acids residues encoded by the 5′-part of the 199 nucleotide intron [29] (Fig. 2F and Supplementary Fig. 2F). Therefore, it was striking to unravel the regulatory mechanism of SRSF1 alternative splicing, which itself is known to regulate the alternative splicing of many target genes. SRSF1 is a critical oncoprotein [30] overexpressed in several cancers including human NSCLC [35]. However, the presence and function of SRSF1-3 are not well characterized yet. Hence, we sought to find the association of the two DUBs in the regulation of SRSF1 alternative splicing for lung cancer progression.

### USP15 and USP4 regulate the mRNA and protein level of SRSF1

**Fig. 3:**
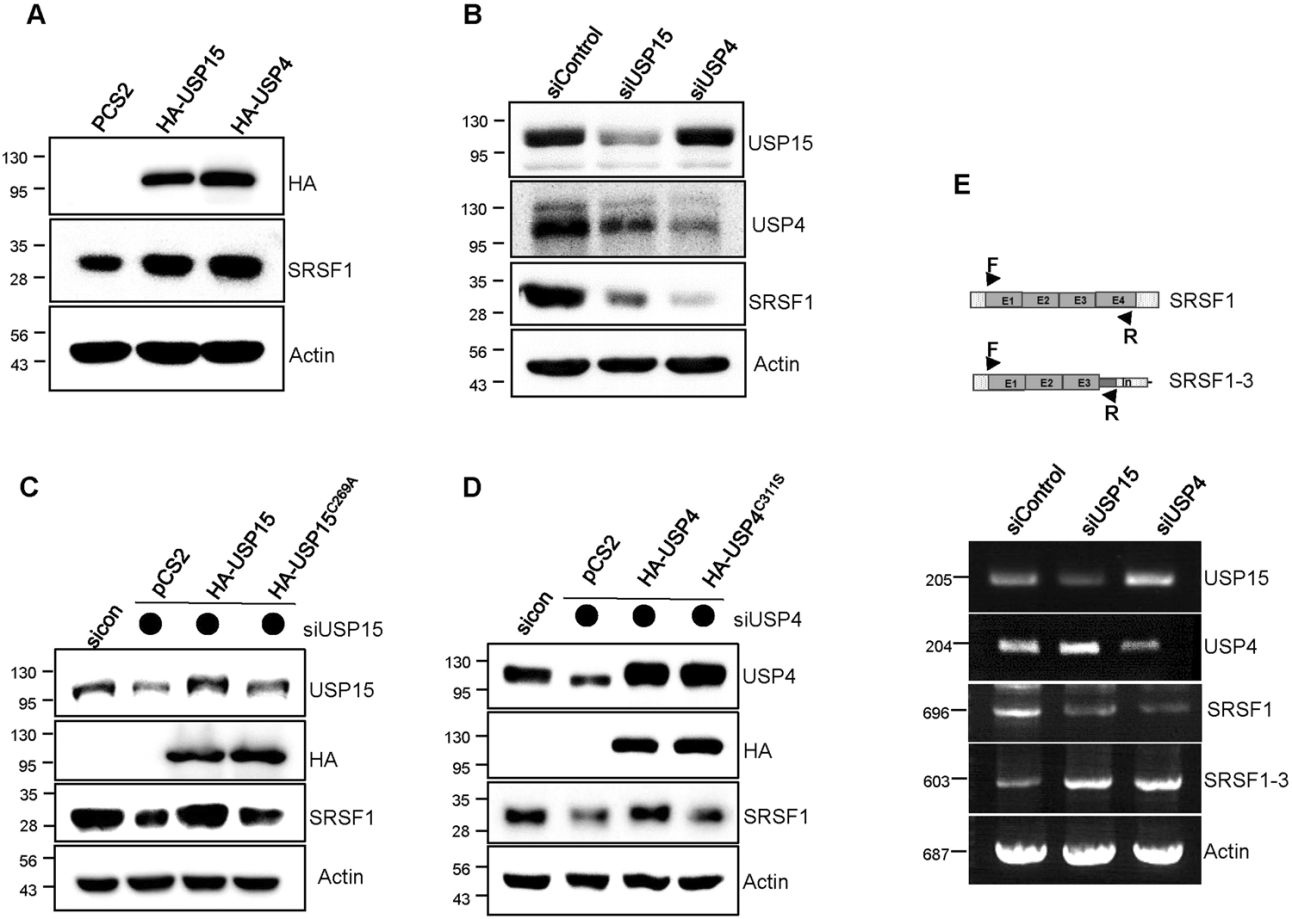
USP15 and USP4 regulate mRNA and protein level of SRSF1. H157 cells were overexpressed with USP15 and USP4 plasmids (**A**) or knockdown with siRNAs (**B**), respectively. After harvesting, proteins were extracted, separated by SDS-PAGE, and the corresponding expression of SRSF1 was detected by western blot. **C** H157 cells were depleted with USP15 siRNA and after 24 h, cells were transfected with either USP15 wild-type or the active site mutants for another 24 h. Proteins were extracted, separated by SDS-PAGE, and detected by western blot using specific antibodies. **D** Knockdown of USP4 was rescued by overexpression of USP4 WT or active site mutant USP4^C311S^ and indicated protein expression was detected by western blot. **E** RNAs were isolated from USP15 and USP4 depleted H157 cells and mRNA expression was detected by RT-PCR with the specific primers designed for SRSF1 as well as SRSF1-3.

To validate the effect of USP15 and USP4 on SRSF1, we checked SRSF1 endogenous expression after overexpression and knockdown of the two DUBs. Overexpression of USP15 and USP4 upregulated SRSF1 (Fig. 3A), while the knockdown caused its downregulation (Fig. 3B). Additionally, the depletion of SRSF1 by USP15 and USP4 knockdown got restored by ectopically overexpressed USP15 or USP4 wild-type, but not by their respective active site mutants (Fig. 3C, D), suggesting that USP15 and USP4 activity is required for SRSF1 regulation. However, in the western blot, only the full-length SRSF1 (*M*_r_ = 33 kDa) was detected, but alternatively spliced lower molecular weight isoform SRSF1-3 was not (data not shown), though the SRSF1 antibody used was N-terminal specific that should detect both isoforms. The non-appearance of SRSF1-3 in protein expression prompted us to check its mRNA level using the specific primers against SRSF1 as well as SRSF1-3. The knockdown of USP15 and USP4 resulted in a decreased SRSF1 mRNA level, with a substantial increase of SRSF1-3 (Fig. 3E), suggesting that USP15 and USP4 regulate the shift of balance between the two alternatively spliced isoforms, SRSF1, and SRSF1-3.

To exclude the possibility that USP15 and USP4 regulate protein level of SRSF1 through deubiquitination, we performed the deubiquitination assay and cycloheximide chase study. As shown in Supplementary Fig. 3A, B, SRSF1 was not deubiquitinated by USP15 or USP4, respectively. In addition, SRSF1 stability was not changed by USP15 or USP4 in the CHX chase study (Supplementary Fig. 3C). Therefore, these data suggest that SRSF1 level is regulated by alternative splicing, not deubiquitination.

### The oncogenic role of SRSF1 is dependent on the alternative spliced isoforms

**Fig. 4:**
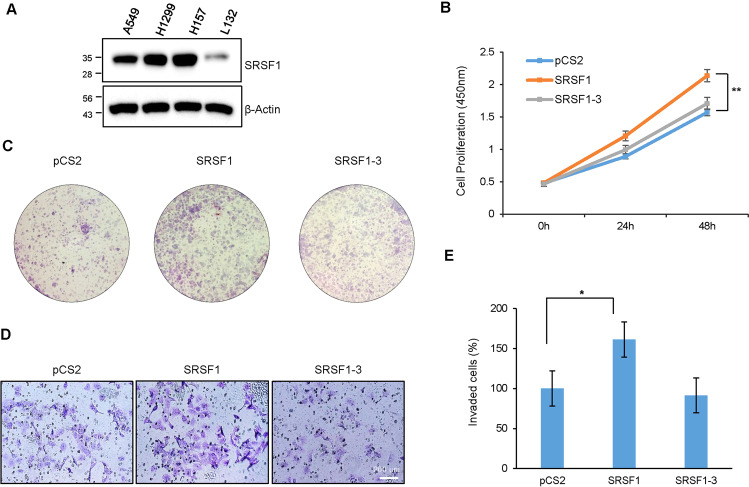
The oncogenic role of SRSF1 is dependent on the alternative spliced isoforms. **A** The expression level of SRSF1 in the lung cancer cells A549, H1299, and H157 was higher compared to the normal lung epithelial cell. **B** H157 cells were transfected with either SRSF1 or SRSF1-3 plasmids and cell proliferation was measured at the indicated time point. **C** SRSF1 or SRSF1-3 transfected H157 cells were reseeded in 30 mm plates following 24 h of post-transfection and continuously cultured until the appearance of visible colonies. The colonies were fixed with methanol, stained with Giemsa stain, and imaged under a light microscope. **D** H157 cells were transfected with SRSF1 or SRSF1-3, harvested after 48 h, and reseeded on a matrigel containing transwells. After 24 h of incubation, invaded cells on the lower chamber of the transwell were fixed, visualized with Giemsa stain, and imaged using a light microscope. **E** The percentage of invaded cells was measured and data from three independent experiments are shown in the bar graph. (*P < 0.05 and **P < 0.01, two-tailed student’s t-test).

Since both DUBs facilitate lung cancer cell proliferation and migration (Fig. 1), and SRSF1 is a decisive candidate regulated by both DUBs (Fig. 3), we subsequently tested SRSF1 endogenous expression to confirm its role in lung cancer progression as well. We found that SRSF1 is upregulated in several lung cancer cells compared to the normal lung epithelial cells (Fig. 4A), similar to the USP15 and USP4 expression patterns. We then cloned SRSF1 and SRSF1-3 in the mammalian expression vector and confirmed the protein expression by western blot (Supplementary Fig. 4A). Following overexpression of SRSF1, we found a significant increase in cell proliferation, while SRSF1-3 did not show any effect (Fig. 4B). Likewise, the colony formation and cell invasion ability of lung cancer cells were enhanced by the overexpression of SRSF1 but not by SRSF1-3 (Fig. 4C–E). The increased invasion by SRSF1 is also confirmed in the A549 cell (Supplementary Fig. 4B). Thus, the enhanced cancer progression by SRSF1 but not by SRSF1-3 suggests that lung cancer cell progression is regulated by the alternative splicing of SRSF1.

### USP15 and USP4 induces cancer cell proliferation via regulating alternative splicing of SRSF1

**Fig. 5:**
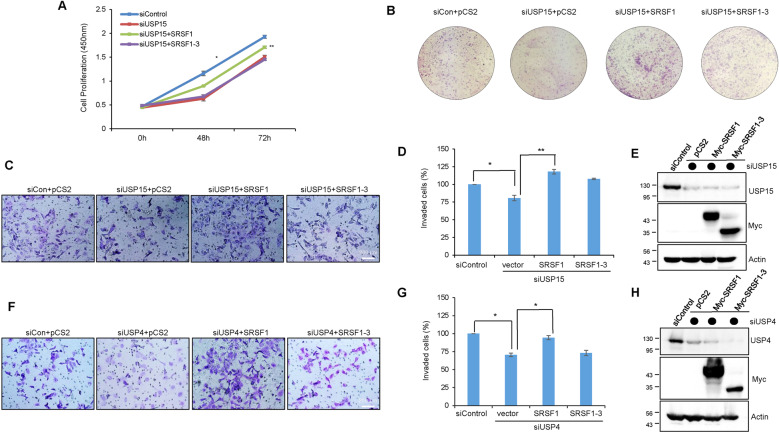
SRSF1, but not SRSF1-3, rescues the USP15 knockdown effect of cancer cell proliferation and invasion. H157 cells were knockdown with USP15 siRNA and 24 h after siRNA transfection, cells were overexpressed with either SRSF1 or SRSF1-3 plasmids. Cell proliferation (**A**), colony formation (**B**), and invasion (**C**, **D**) were detected as described previously. **E** The knockdown efficiency of USP15 and subsequent overexpression of SRSF1/SRSF1-3 were confirmed by western blot. H157 cells were transfected with USP4 siRNA and rescued with either SRSF1 or SRSF1-3 expression plasmids and cell invasion was imaged (**F**) and measured (**G**). **H** USP4 knockdown and SRSF1/SRSF1-3 overexpression were confirmed by western blot.

Next, we examined whether the cancer progression ability of USP15 and USP4 is allied with SRSF1 alternative splicing. The depletion of USP15 resulted in a substantial reduction of cell proliferation, colony formation, and invasion ability as consistent. But strikingly, the USP15 depleted reduction of cell proliferation and colony formation was restored by the overexpression of SRSF1 (Fig. 5A, B). Also, the subsequent overexpression of SRSF1 restored the decreased invasion by USP15 (Fig. 5C, D) and USP4 (Fig. 5F, G) knockdown in H157 cells, as well as in A549 cells (Supplementary Fig. 5A, B). However, SRSF1-3 was unable to restore any of these depletion effects. The knockdown of USP15 and USP4 and subsequent overexpression of SRSF1 or SRSF1-3 were confirmed by western blot (Fig. 5E, H), respectively. Collectively, we concluded that the oncogenic property of USP15 and USP4 is associated with SRSF1 that shows a parallel regulatory effect in lung cancer cell while the alternatively spliced isoform SRSF1-3 lacks such oncogenic functions.

### SRSF1-3 undergoes nonsense-mediated mRNA decay

**Fig. 6:**
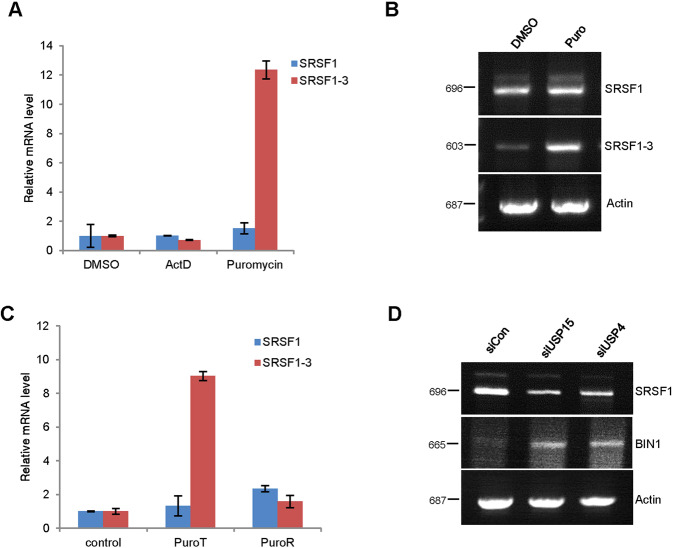
SRSF1-3 undergoes nonsense-mediated mRNA decay. **A** H157 cells were transfected with either SRSF1 or SRSF1-3 expression plasmids and after 24 h of transfection, cells were treated with the transcription inhibitor actinomycin D (5 nM) or the translational/ NMD inhibitor puromycin (100 μg/ml) with control DMSO for 4 h. After harvest the treated cells, RNA was isolated, synthesized the first strand of cDNA, and subjected to qPCR using 2 × SYBR Green*/*RoxMasterMix. The experiments were performed in triplicate for each data point, and the relative quantification in gene expression was determined with the 2^−*∆∆*Ct^ method. **B** The SRSF1 or SRSF1-3 transfected H157 cells were treated with puromycin or control DMSO and RNA isolation was done as in (**A**). The cDNA was synthesized with M-MLV reverse transcriptase using oligo-dt and the PCR reaction was run with 94 °C denaturations and 57 °C annealing temperature for 35 cycles. The reaction samples were loaded in 1.5% agarose gel to visualize the RNA expression of SRSF1 and SRSF1-3 with β-actin as a loading control. **C** H157 cells were transfected with either SRSF1 and SRSF1-3 and treated with 100 μg/ml of puromycin (PuroT). After 4 h of treatment, cells were washed two times with 1×PBS and add fresh media to release puromycin (PuroR) and incubated for another 8 h before harvest. The relative expression of SRSF1 and SRSF1-3 mRNAs were detected by qPCR as described in (**A**). **D** H157 cells were transfected with USP15 siRNAs for 48 h and RNA was isolated after harvest the cells. The cDNA was synthesized from the RNA template and the PCR reaction was run as described in (**B**). Samples were loaded in 1.5% agarose gel to visualize the expression of the SRSF1 and its target gene BIN1, while β-actin was used as the loading control.

As SRSF1-3 contains the premature termination codon (PTC) in its C-terminal retained intron region (Supplementary Fig. 2F), we examined whether SRSF1-3 is regulated by NMD considering the previous report that mRNAs harboring PTCs or nonsense codons are degraded by NMD to control the quality and fidelity of gene expression [36, 37]. Following the previous report demonstrating that the treatment with the translational inhibitor puromycin suppresses NMD [38], we treated cells with puromycin and detect the mRNA level of SRSF1-3. As shown in Fig. 6A, B, puromycin treatment significantly increased SRSF1-3 mRNA level compared to the control, while SRSF1 mRNA was not altered considerably. Again, the subsequent release of puromycin triggered SRSF1-3 degradation with the decreased mRNA expression (Fig. 6C), suggesting SRSF1-3 expression is regulated by NMD. Finally, we examined whether USP15 and USP4 mediated regulation of SRSF1 affects the SRSF1 downstream genes as well. SRSF1 mediated alternative splicing of several oncogenes and tumor suppressors regulate the downstream pathways affecting cell proliferation and apoptosis [31, 39]. BIN1 is a well-recognized tumor suppressor among the SRSF1 target genes, which is lost in several NSCLC, and restored expression of BIN1 can suppress malignant phenotypes [40]. We observed an increased expression of BIN1 through SRSF1 downregulation by USP15 and USP4 knockdown (Fig. 6D), which may, in turn, inhibits the metastatic ability of lung cancer cells. Collectively, our current findings highlight the vital regulatory mechanism of SRSF1 alternative splicing in lung cancer cell, which strengthens the potentiality of SRSF1 as a diagnostic marker.

### USP15 expression contributes to lung adenocarcinoma prognosis

**Fig. 7:**
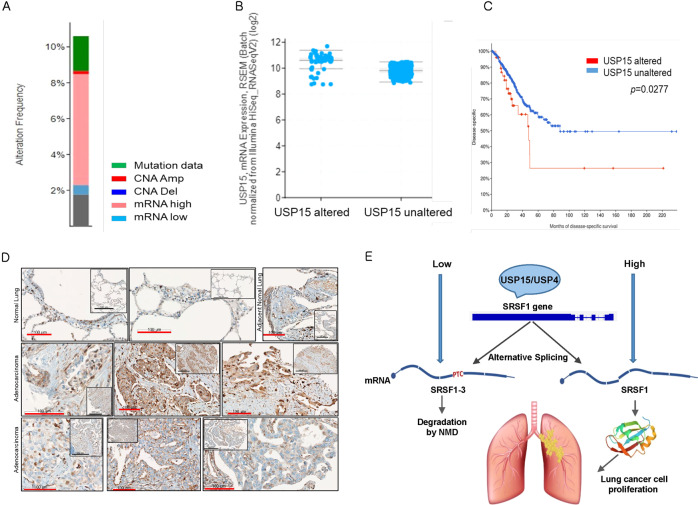
USP15 alteration is closely related to LUAD survival. **A** Alteration frequency of USP15 in LUAD of TCGA PanCancer Atlas. The USP15 alteration frequency was obtained in LUAD patients (TCGA, PanCancer Atlas) by using cBioportal. **B** The expression of USP15 at mRNA level in tissues was compared in terms of USP15 alteration. One group with USP15 alteration showed higher expression than the other group without USP15 alteration (*p* = 1.39 × 10^−9^) of LUAD (TCGA PanCancer Atlas, cBioportal). **C** Disease-specific survival of LUAD (TCGA PanCancer Atlas, cBioportal) showed poor prognosis in the group (*n* = 53) with USP15 alteration comparing to that in the group without USP15 alteration (*n* = 470). *p* = 0.0277. **D** USP15 expression increases tumor progression in lung adenocarcinoma. Lung tissues from a human lung adenocarcinoma tissue array (US Biomax, Inc.; LC10013c) were stained with hematoxylin and anti-USP15 antibody, then detected using DAB. Inserts show low magnification of tissue images. Red scale bar: 100 μm, Black scale bar: 300 μm, respectively. **E** Schematic presentation of USP15 and USP4 mediated lung cancer progression by regulating SRSF1 alternative splicing. High USP15 and USP4 upregulate SRSF1 and induce lung cancer phenotypes through enhancing cell proliferation and invasion. Conversely, low USP15 and USP4 upon depletion decrease SRSF1 with a substantial increase of the alternative isoform SRSF1-3, which is degraded by NMD and thus lack the cancer progression effects.

For the clinical significance of USP15 in lung cancer, we checked the USP15 correlation with LUAD development and the patient’s survival by analyzing the TCGA LUAD data, especially PanCancer Atlas using cBioportal [36, 37, 41]. Interestingly, the alteration frequency of USP15 was over 10% in LUAD. Of these, 60% alteration is related to increased USP15 expression, such as copy number amplification and high mRNA expression (Fig. 7A). In addition, the group with USP15 alteration showed higher USP15 expression at mRNA level than in the other group without USP15 alteration (Fig. 7B). Furthermore, patients with USP15 alteration (*n* = 53) showed significantly poorer disease-specific survival (Fig. 7C), implying that USP15 expression correlates with LUAD development and progression in patients.

Next, we further investigated if USP15 might be expressed in normal and tumor tissues and if USP15 might be increased in tumor tissues of lung adenocarcinoma. To do this, we obtained tissue arrays from Biomax Inc., which contain more than forty malignant adenocarcinomas, adjacent normal tissues, and four normal lung tissues. Stained tissues with hematoxylin and specific antibody against USP15 were examined by professional pathologists (Fig. 7D). Interestingly, USP15 was observed through all tissues on tissue arrays. In normal lung tissues, USP15 was localized in bronchial epithelium normal lung, and some stromal cells and macrophages of alveolar airway epithelium (Fig. 7D, top left). In adjacent normal tissue, bronchial epithelial area and some stromal cells were stained with USP15 antibody strongly more than normal lung tissues. We observed USP15 expression in adenocarcinoma tissues of lung clearly and the expression level of USP15 was variable but generally increased through tumor tissues (Fig. 7D, middle and bottom). The localization of USP15 in tumor tissues was also nucleus, cytosol, or membrane. Taken together, these results suggest upregulation of USP15 expression correlates with LUAD development in vivo.

## Discussion

Some DUBs have both oncogenic and tumor-suppressive functions by regulating multiple substrates in different cellular pathways. However, their roles in the alternative mRNA splicing, particularly of cancer-associated genes, are not yet well understood and need further attention. Previously, we showed that the spliceosomal dynamics require USP15 and USP4 to deubiquitinate the U4 snRNP components PRP31 and PRP3, respectively, for efficient mRNA splicing of chromosome segregation associated genes such as Bub1 or α-tubulin [1, 34]. In this study, we sought to find whether these two DUBs mediate cancer cell proliferation and migration through splicing regulation of other associated genes. Using RNA sequencing analysis, we acquired a vast list of alternatively spliced genes regulated by USP15 and USP4 with diverse cellular functions like mitotic regulation, cell cycle, transcription, protein metabolism, splicing, DNA repair, apoptosis, and so on (Fig. 2A–C and Supplementary Fig. 2C–E). Among them, we predominantly focused on SRSF1, which itself is a recognized splicing regulator of other target genes and involved in several malignancies. Therefore, examining the splicing regulation of the splicing factor SRSF1 by USP15 and USP4 in cancer progression was our particular interest. A very recent study suggested that USP42 could be involved in the regulation of multiple mRNA splicing by driving the nuclear speckle separation and incorporation of the spliceosome component [42]. Another recent report showed that USP39 maintains the efficient splicing of the oncogenic transcription factor high mobility group AT-hook (HMGA2) by promoting its splicing efficiency rather than exon skipping. Moreover, USP39 was found to be overexpressed in high-grade serous ovarian carcinoma (HGSOC) and promotes ovarian cancer malignancy, thereby involved in the poor prognosis of patients with HGSOC [43]. Similarly, our current study found an altered USP15 expression in lung adenocarcinoma, which is correlated to LUAD development and contributes to poorer disease-specific survival (Fig. 7A–C).

More than a shred of evidence is in favor of the proto-oncogenic role of SRSF1 indicates that it is associated with different cancers and regulates cell proliferation and apoptosis [30, 31]. SRSF1 regulates the alternative splicing and functions of the target genes involved in cancer progression [32]. For example, SRSF1 regulates the alternative splicing of the tumor suppressor BIN1, which interacts with the proto-oncogene MYC to inhibit its proliferative activity [31], while SRSF1 overexpression endorses BIN1 exon12a inclusion that lacks tumor-suppressor activity due to reduced binding with MYC [44]. SRSF1 also impairs BIM-mediated apoptosis by promoting the expression of BIM isoforms lacking the pro-apoptotic activity [31]. Additionally, the alternative splicing of the oncogene MDM2 is negatively regulated by SRSF1 under DNA damage and arises the isoform MDM2-ALT1 with the tumorigenic ability [45]. SRSF1 regulates the alternative splicing of many other cancer-related genes such as BCLX, MCL1, CASP2, CASP9, CCND1, RPS6KB1, RAC1, RON, MKNK2, etc. which are reported to involve in cell proliferation, apoptosis, or cell cycle progression [46]. Recently SRSF1 is found to regulate the alternative splicing of the serine-threonine kinase DBF4B that plays a vital role in promoting tumorigenesis in colon cancer cells [47]. Besides regulating the target genes, SRSF1 itself is dysregulated in several malignancies therefore considered as a proto-oncogene [30]. SRSF1 autoregulates its own transcript by alternative splicing and the expression of unproductive isoforms is delimited by nonsense-mediated decay [28, 48, 49]. SRSF1 expression is also shown to be regulated by ubiquitination [50]. However, only a few reports have demonstrated their detailed regulatory mechanism to date. In particular, there is rare evidence showing the isoform-specific functional variance of SRSF1. Here we found the enhanced oncogenic ability by SRSF1 in lung cancer cell, while the isoform SRSF1-3 was functionally inactive (Fig. 4). Afterward, we confirmed the functional deficiency of SRSF1-3 is due to its enhanced degradation by NMD (Fig. 6) because of containing a premature stop codon in its C terminal intron sequences (Supplementary Fig. 2F). Therefore, the functional outcome of SRSF1 is achieved by its alternative splicing, and the deubiquitinating enzymes USP15 and USP4 regulate this process (Fig. 5).

In summary, we found that the deubiquitinating enzymes USP15 and its close paralog USP4 regulate alternative splicing of SRSF1 resulting in the isoform-specific functions in lung cancer cell. In low USP15 and USP4 upon depletion, SRSF1 is down-regulated with substantial retention of the C-terminal intron sequences recognized as an altered isoform SRSF1-3, which is degraded by NMD and thus do not show any effects on cancer cell phenotype. On the other hand, SRSF1 is upregulated by high USP15 and USP4 that enhance cell proliferation and invasion (Fig. 7D). Additionally, the depletion effect of USP15 and USP4 is restored by SRSF1 but not by SRSF1-3. Moreover, analysis of the alteration frequency of USP15 in TCGA LUAD data showed that increased USP15 expression contributes to LUAD development. Furthermore, patients with USP15 alteration showed significantly lower disease-specific survival. From tissue arrays, we observed the expression of USP15 in lung adenocarcinomas. Some adenocarcinoma tissues expressed USP15 strongly, whose location in cells were variable in nucleus, cytosol, or membrane. We are further investigating the relationship between cellular location of USP15 and tumor malignancy during lung adenocarcinoma progression. Our current study provides the first evidence of DUB-mediated regulation of SRSF1 alternative splicing that controls its oncogenic properties in lung cancer cell. Collectively our data provide important evidence for the global regulation of critical splicing factors and alternative splicing by the deubiquitinating enzymes in lung cancer cell proliferation and migration.

## Materials and methods

### Cell culture and transfections

Non-small cell lung cancer (NSCLC) H157, H1299, A549, and the lung epithelial L132 cells were maintained in RPMI medium supplemented with 10% heat-inactivated fetal bovine serum, penicillin (10 U/mL), and streptomycin (100 μg/mL). Three different USP15 siRNA sequences were used to knockdown, siUSP15_1 (5′-CUAUGGAAAUGAUGAAGUU-3′), siUSP15_2 (5′-AGGAAUGAGAGGUGAAAUA-3′), and siUSP15_3 (5′-GCAGAUAAGAUGAUAGUUA-3′). USP4 targeting siRNA sequences were siUSP4_1 (5′- CGAAGAAUGGAGAGGGAACA -3′), siUSP4_2 (5′-GACAUGUACAAUGUGGGUGAA-3′), and siUSP4_3 (5′- CAGUUGAGCAAGCUAGACAAC-3′), while sequence 5′-CCUACGCCACCAAUUUCGU-3′ was used as a negative control. All siRNAs and plasmids were transfected using lipofectamine 2000 and transfection reagent respectively, according to the manufacturer’s instructions.

### Establishment of USP15 and USP4 knockdown H157 stable cells lines

USP15_1 and USP4_1 siRNA sequences for the depletion of the two respective DUBs were cloned into the pLPCX retroviral vector and the presence of recombinant vectors was confirmed via DNA sequencing. USP15/USP4 siRNA retroviral vectors were then transfected into HEK293T cells with the packaging plasmids Gag-Pol and the envelope plasmids VSV-G. 48 h after transfection, the supernatant was collected, filtered, and infected to H157 cells. The infected cells were selected with 0.5 μg/ml puromycin for up to 1–2 weeks, expand the selected single colones to a higher scale, and the gene depletion was confirmed by western blot. As a control, empty retroviral stable cell lines were produced with the same protocol.

### RNA sequencing analysis and data processing

Total RNAs were extracted from the USP15 and USP4 knockdown H157 stable cells together with the control and RNA sequencing was processed and analyzed (Macrogen Inc.). The raw reads were preprocessed from the sequencer to remove low quality and adapter sequence before analysis and aligned the processed reads to the Homo sapiens (GRCh37) using HISAT v2.1.0 [51]. The reference genome sequence of Homo sapiens (GRCh37) and annotation data were downloaded from the NCBI and the transcript assembly of known, novel, and alternative splicing transcripts was processed. The expression abundance of the transcript was calculated as read count or FPKM value per sample. The RNA-seq data were further analyzed (eBiogen Inc.) using Mixture of Isoforms (MISO) software to detect the alternatively spliced gene patterns and to identify the novel splicing events. To perform gene-annotation enrichment analysis, the genes were selected and input to DAVID functional annotation tool (https://david.ncifcrf.gov/summary.jsp).

### Cloning

USP15 WT (isoform 2) used in this study is the same plasmid described in our previous study [1]. SRSF1 full length and the alternatively spliced variant SRSF1-3 genes were purchased from gene banks (https://genebank.kribb.re.kr), amplified by PCR, and cloned into the Asc1 and Fse1 restriction site of pCS2 expression vector incorporating Myc tag on the N-terminus.

### Cell proliferation, migration, and invasion assay

Cells were transfected with plasmids or siRNA as indicated and incubated for 24–72 h. Cell viability was determined by the EZ-Cytox cell viability assay kit at 450 nm using the microplate reader. For migration assay, transfected cells were grown until 100% confluency and make a vertical wound down through the cell monolayer using a pipette tip. An initial picture was taken following wound generation, continuously culture the plate, and imaged at regular intervals until complete healing of the wound. For invasion assay, transfected cells were harvested after 48 h and reseeded on the transwell (Falcon, 24 well with the format of 8 μm pore) containing the pre-gelled matrix (BD matrigel matrix, 356234). Put each transwell in the individual well of a 24 well plate and after 24 h, invaded cells on the lower surface of the transwell were fixed, stained with Giemsa, and visualized and pictured using a light microscope.

### Colony formation assay

H157 cells were transfected with indicated plasmids or siRNA and after 24 h, cells were harvested and reseeded into 35 mm plates (1 × 10^3^/dish) and cultured continuously until the visible colonies appeared. The colonies were fixed with methanol, stained with Giemsa stain, and pictured under a light microscope (equipped at Ewha Drug Development Research Core Center).

### RT-PCR and qPCR analysis

RNA was isolated with the RNeasy^®^ mini kit (Qiagen) and synthesized the first strand of cDNA with M-MLV reverse transcriptase (Bioneer) using oligo-dt (Bioneer) as primers. PCR reaction was run with 94 °C denaturations and 57 °C annealing temperature for 35 cycles. The primers used to amplify the genes were, USP15 (F-AAGGTCAACTCACGGGACAC, R-CCTCGGCAGCATTTTCATCA), USP4 (F-TACCGAGGCGTGGAATAAAC, R-GATGGTTGCAATGGTGTCTG), SRSF1 (F-ATGTCGGGAGGTGGTGTGAT, R-TCTGCTTCTCCTTGGGGAGT), SRSF1-3 (F-ATGTCGGGAGGTGGTGTGAT, R-AATTCCACTGTTAAGACCAC) while β-actin (F-TGACGGGGTCACCCACACTG, R-CTAGAAGCATTTGCGGTGGA) were used as a loading control. For qPCR, isolated RNA was reverse transcribed using the Thermo Scientific DyNAmo cDNA Synthesis Kit with a random hexamer primer and the reactions were run with 2×SYBR Green*/*RoxMasterMix (Thermo Scientific), 200 nM primers, and 100 ng of RNA. All experiments were done in triplicate, and the relative quantification in gene expression was determined with the 2^−*∆∆*Ct^ method.

### Western blot analysis

Cells were harvested, washed twice with ice-cold PBS, lysed in lysis buffer containing 50 mM HEPES (pH 7.5), 150 mM NaCl, 1.5 mM MgCl_2_, 5 mM KCl, 0.1% Tween-20, 2 mM DTT, and protease inhibitor cocktail (Roche), incubated on ice for 10 min and centrifuged at 15,000 × *g* for 10 min. Supernatants were collected as whole cell lysates and loaded for SDS/PAGE analysis. The antibodies used were, USP15 (Bethyl Laboratories Inc.), USP4 (Bethyl Laboratories Inc.), β-actin (Santa Cruz Biotechnology Inc.), Myc (Santa Cruz Biotechnology), HA (Santa Cruz Biotechnology), Actin (AB frontier), and SRSF-1 (Santa Cruz Biotechnology Inc.).

### Statistical analysis

The statistical analysis of experimental data was carried out using the Student’s t-test at *p* < 0.05. Data are presented as mean ± S.D.

### Immunohistochemistry (IHC) analyses

USP15 staining of tissue arrays (US Biomax, Inc., Rockville, MD, USA; http://www.biomax.us/, slide LC10013c) was conducted by the National Cancer Center Animal Sciences Branch. Lung tissue in the tissue microarrays was stained with hematoxylin and mouse anti-USP15 (1:200) antibody and detected with 3,3′-diaminobenzidine (DAB). The stained tissues on the tissue microarray were examined by professional pathologists for USP15 expression in tumor tissues of array and were digitized at 20 × magnification using the Vectra^®^ Polaris^TM^ (Akoya Biosciences Inc., USA) equipped with a clinical grade RGB camera [52]. Loss of zinc-finger protein 143 contributes to tumor progression by interleukin-8-CXCR axis in colon cancer [52]. Images from the slide not stained with primary antibody were used as a negative control.

## Supplementary information

Supplementary Figure 1

Supplementary Figure 2

Supplementary Figure 3

Supplementary Figure 4

Supplementary Figure 5

uncropped original data

Supplementary Figure Legend

## Data Availability

The datasets generated and/or analyzed during the current study are available from the corresponding author on reasonable request.
