# Supplementary figures and images for: USP15 and USP4 facilitate lung cancer cell proliferation by regulating the alternative splicing of SRSF1
Source: Cell Death Discov. 2022 Jan 13;8:24. doi: 10.1038/s41420-022-00820-0 (PMC8758713; doi:10.1038/s41420-022-00820-0)

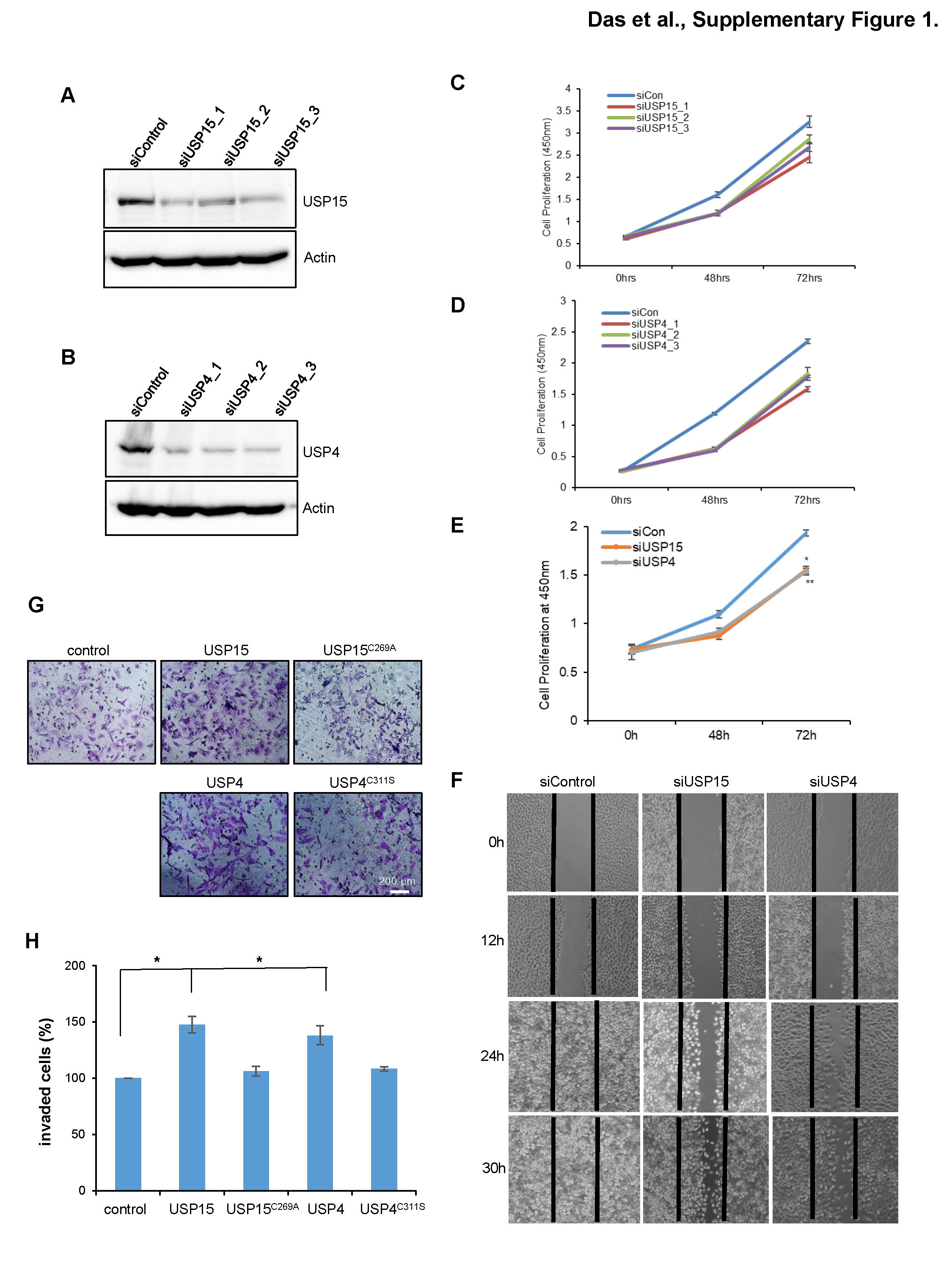

Supplement: Supplementary file 1 — Supplementary Figure 1 [file 41420_2022_820_MOESM1_ESM.jpg]

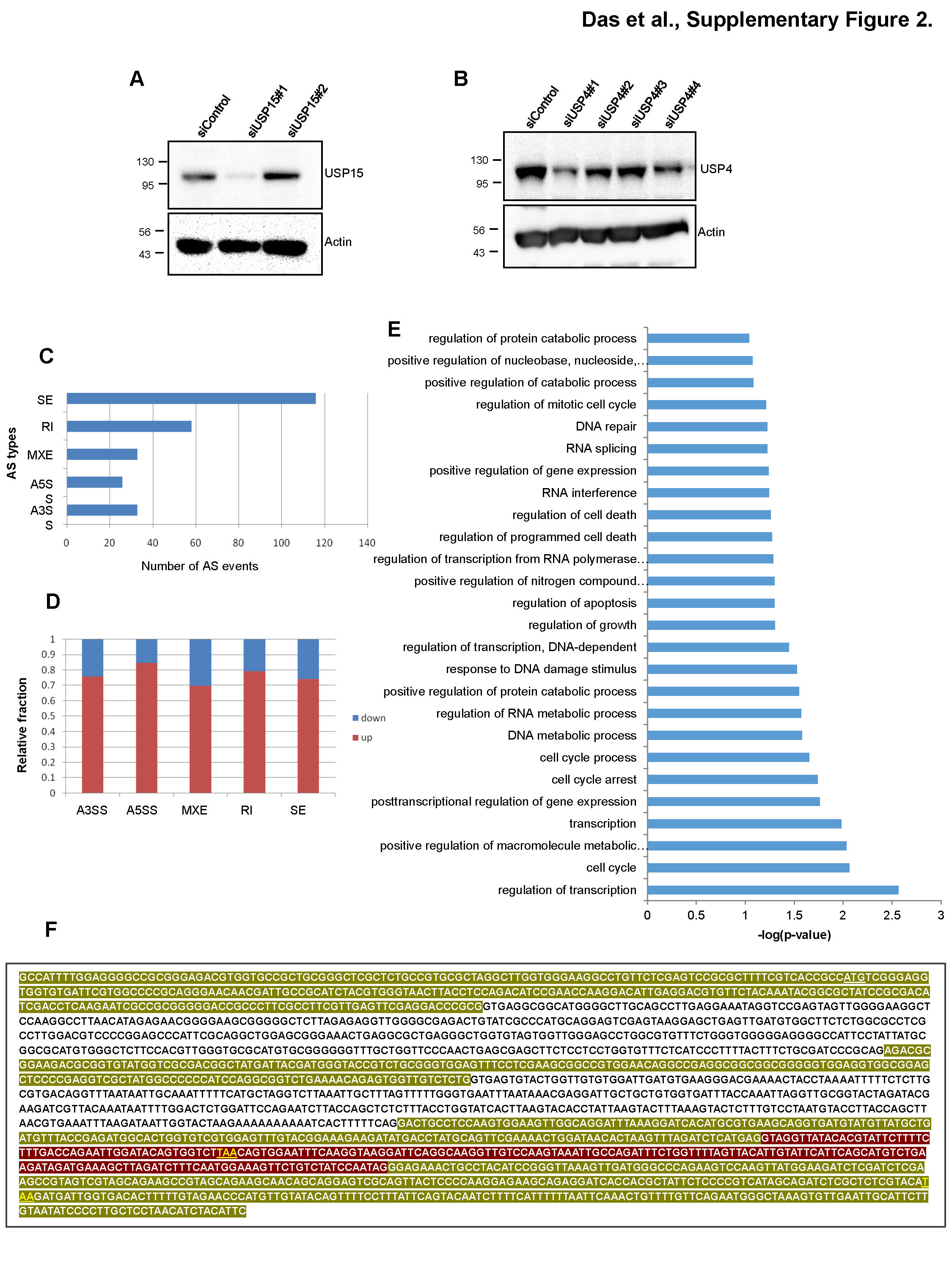

Supplement: Supplementary file 2 — Supplementary Figure 2 [file 41420_2022_820_MOESM2_ESM.jpg]

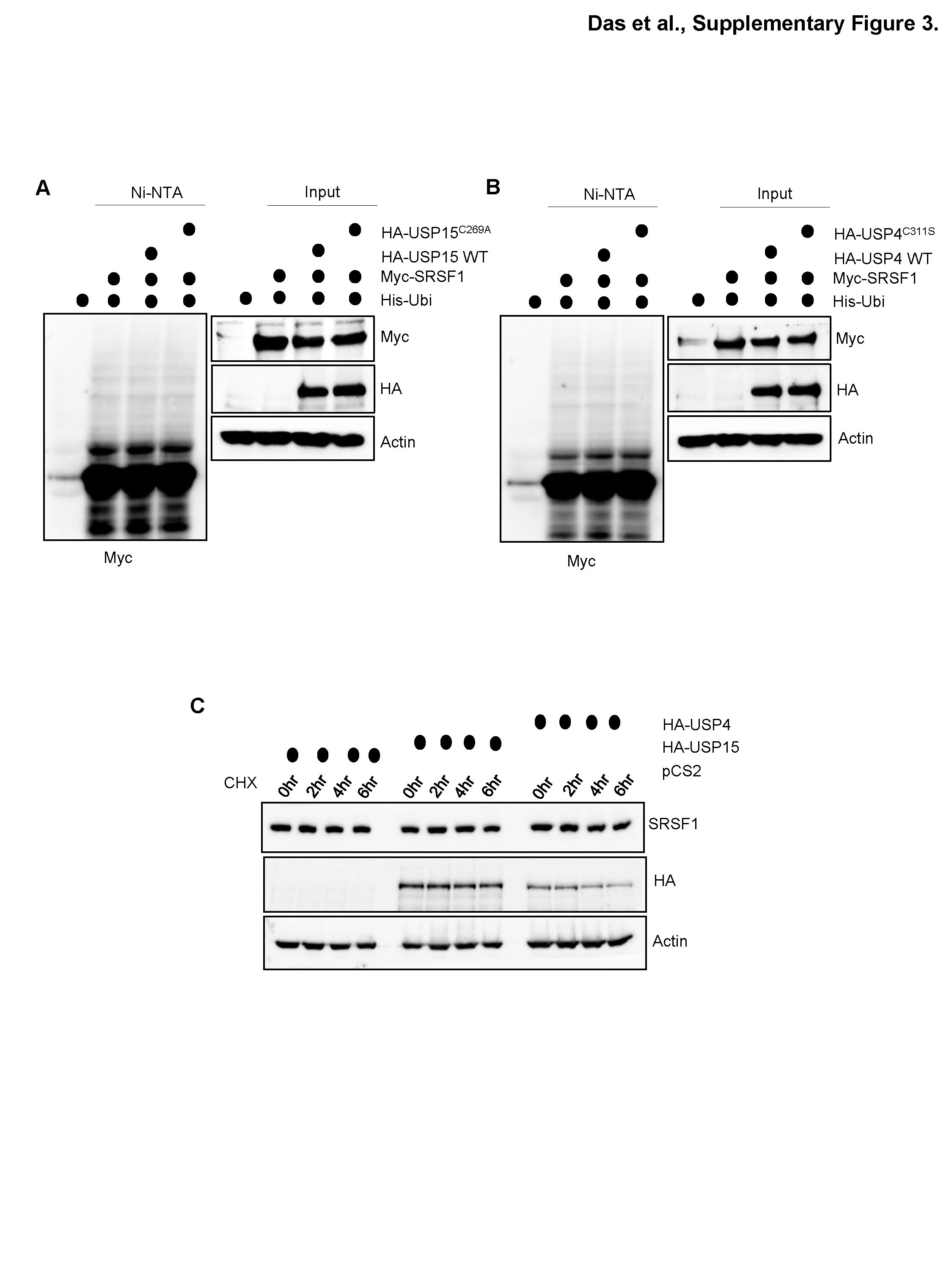

Supplement: Supplementary file 3 — Supplementary Figure 3 [file 41420_2022_820_MOESM3_ESM.jpg]

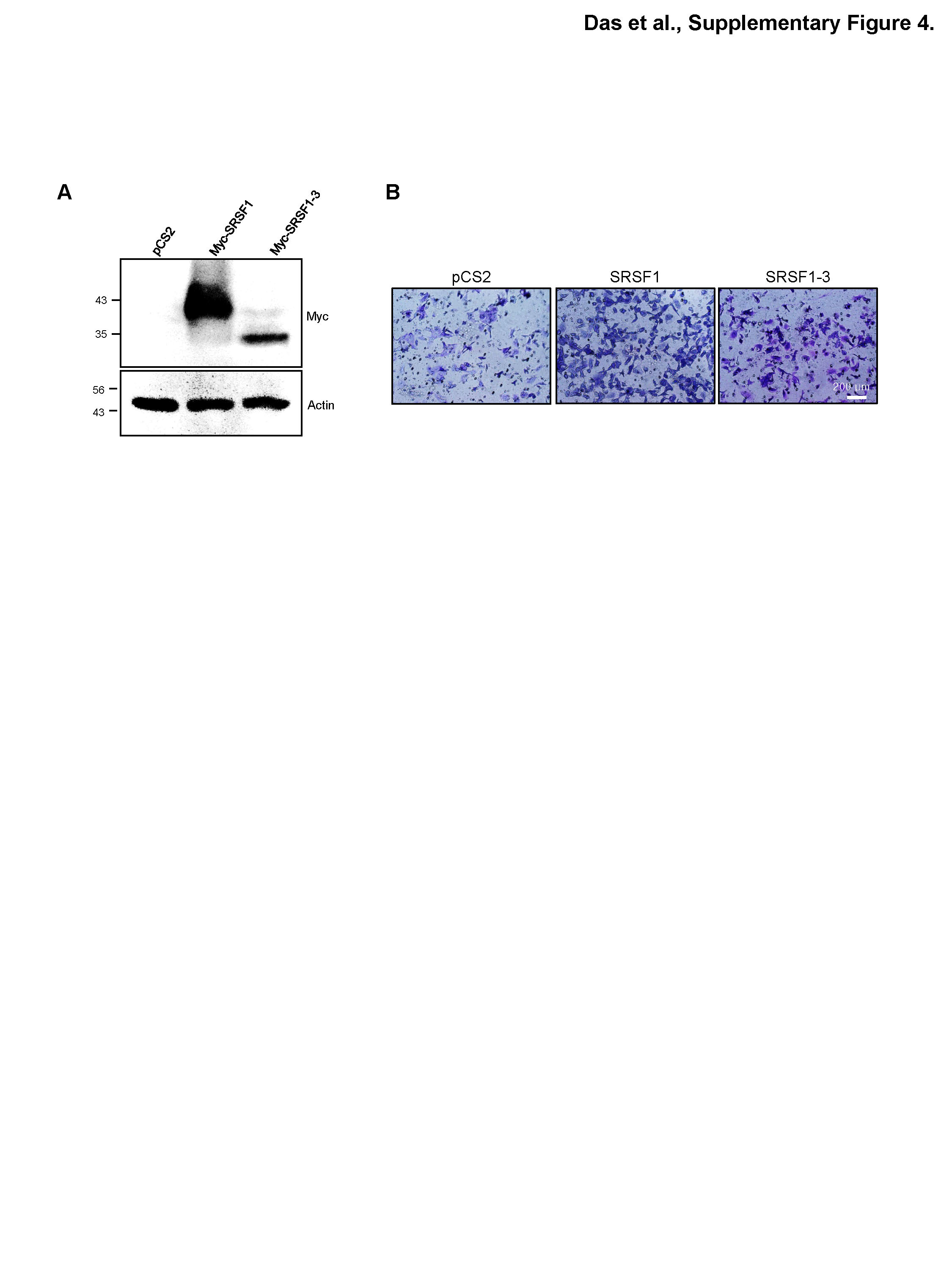

Supplement: Supplementary file 4 — Supplementary Figure 4 [file 41420_2022_820_MOESM4_ESM.jpg]

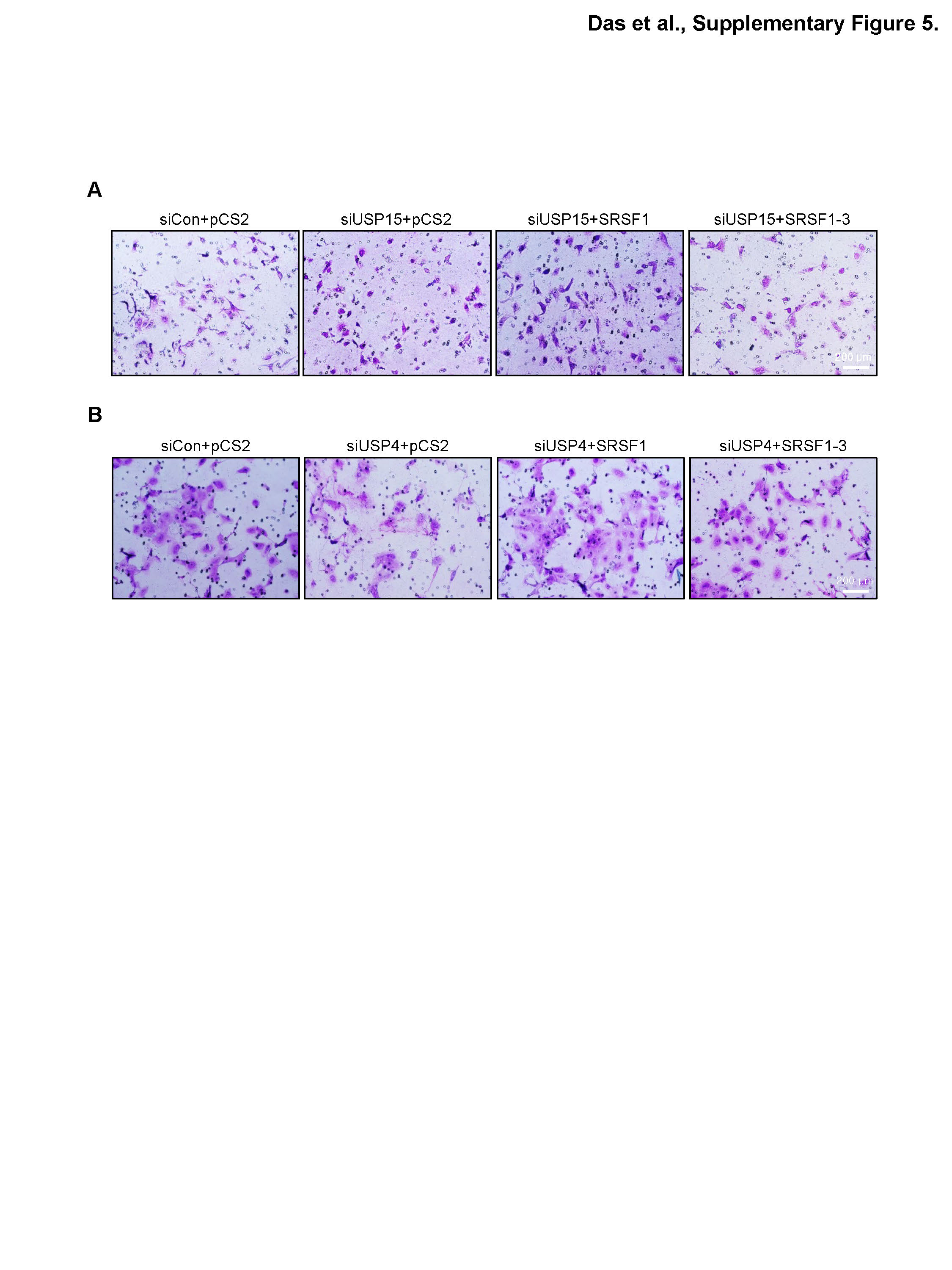

Supplement: Supplementary file 5 — Supplementary Figure 5 [file 41420_2022_820_MOESM5_ESM.jpg]

**A**

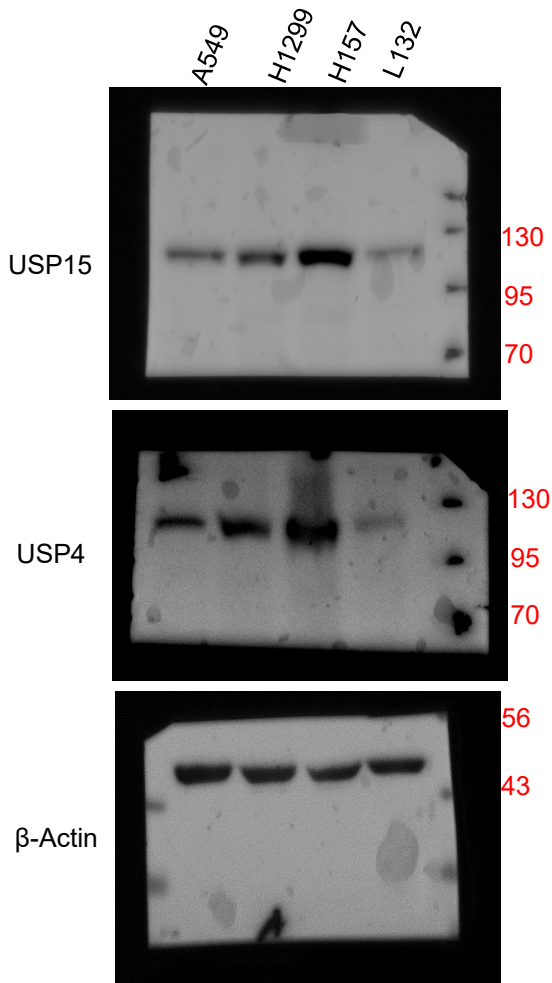

**B**

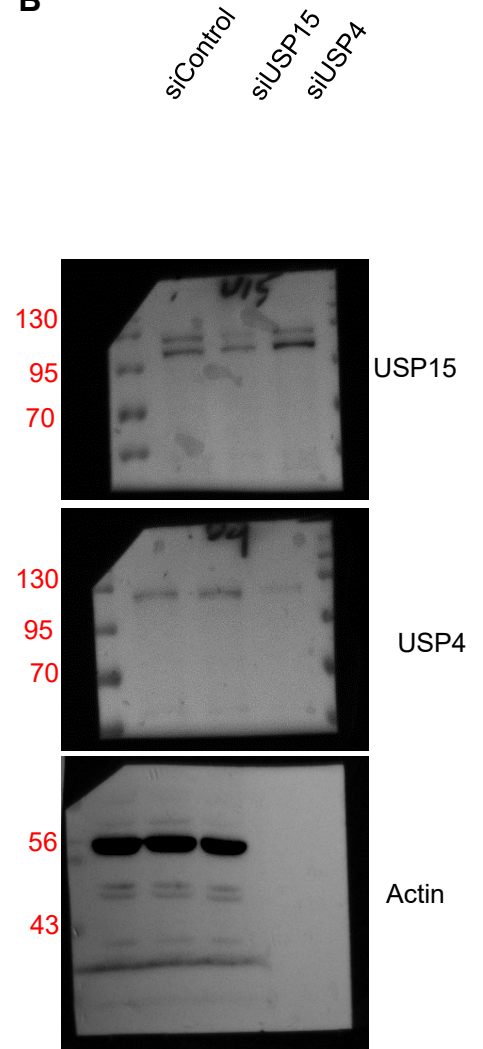

**H**

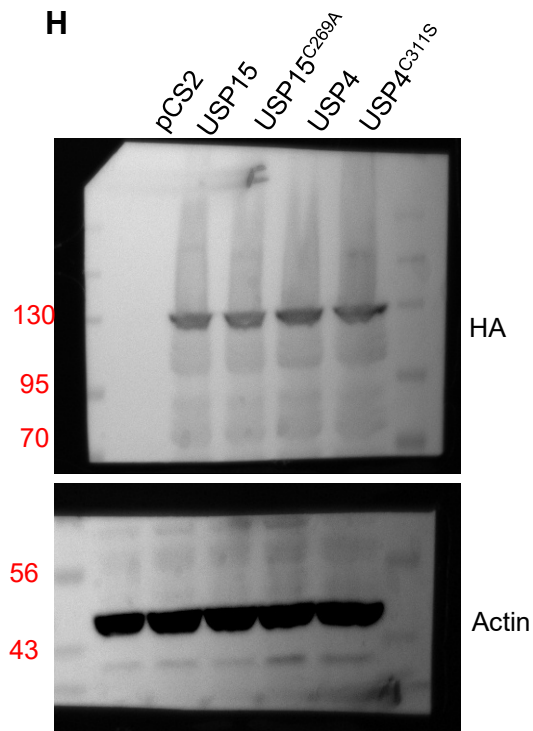

**A**

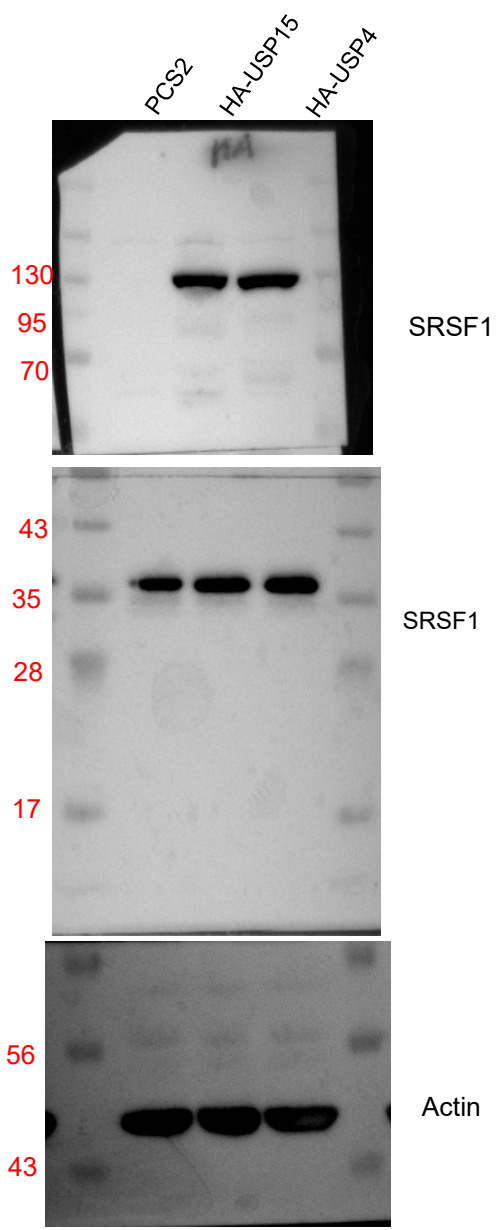

**B**

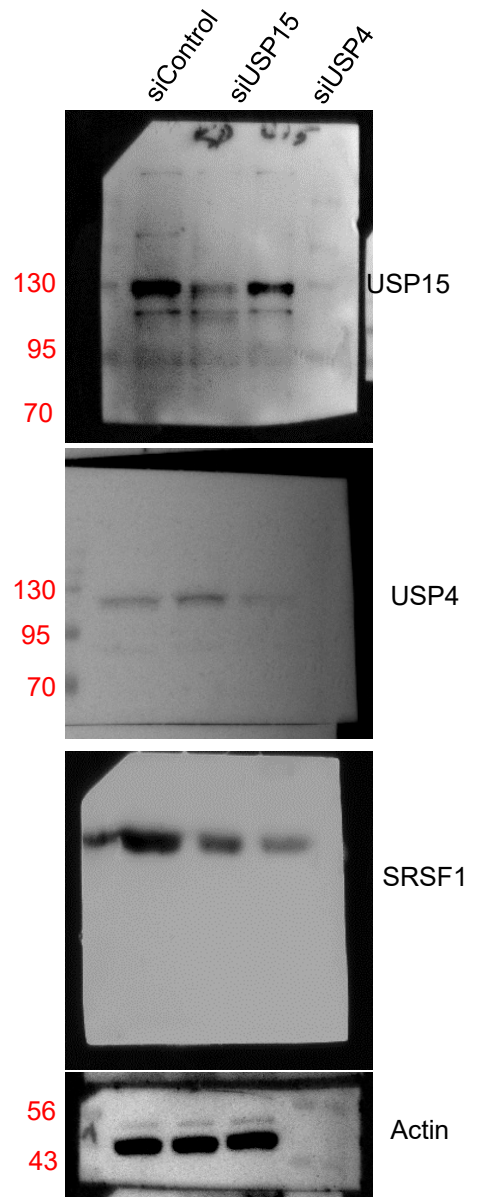

C

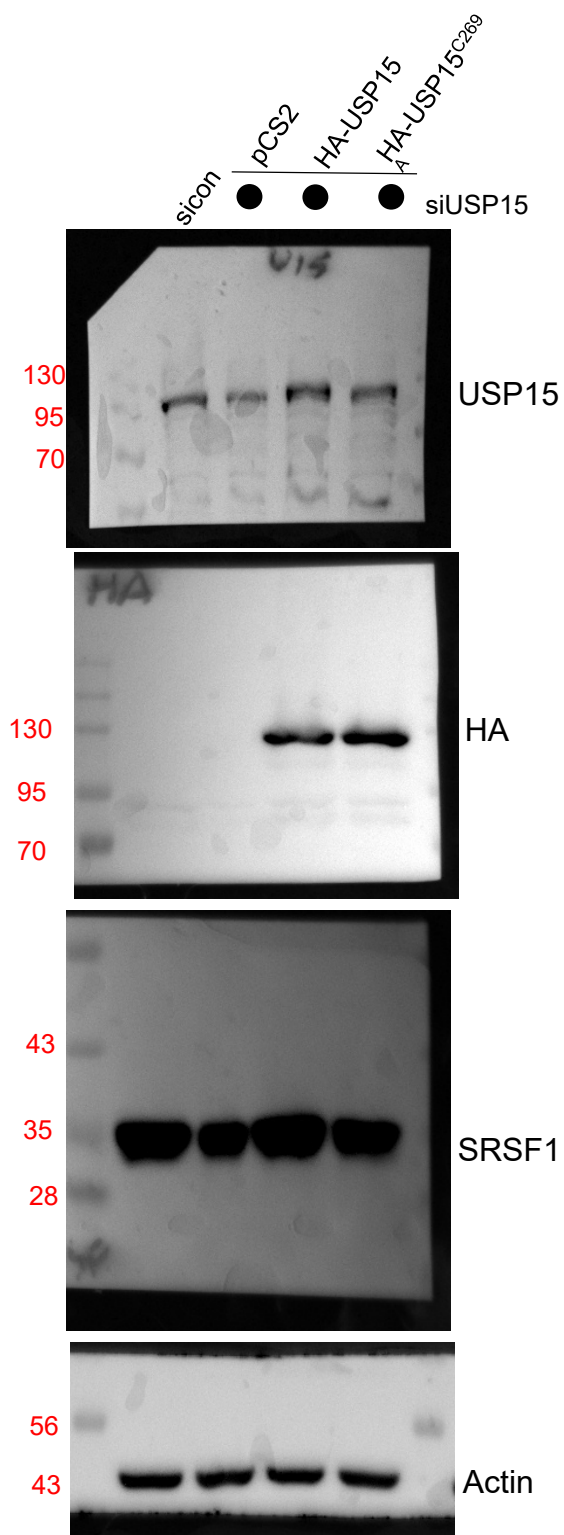

D

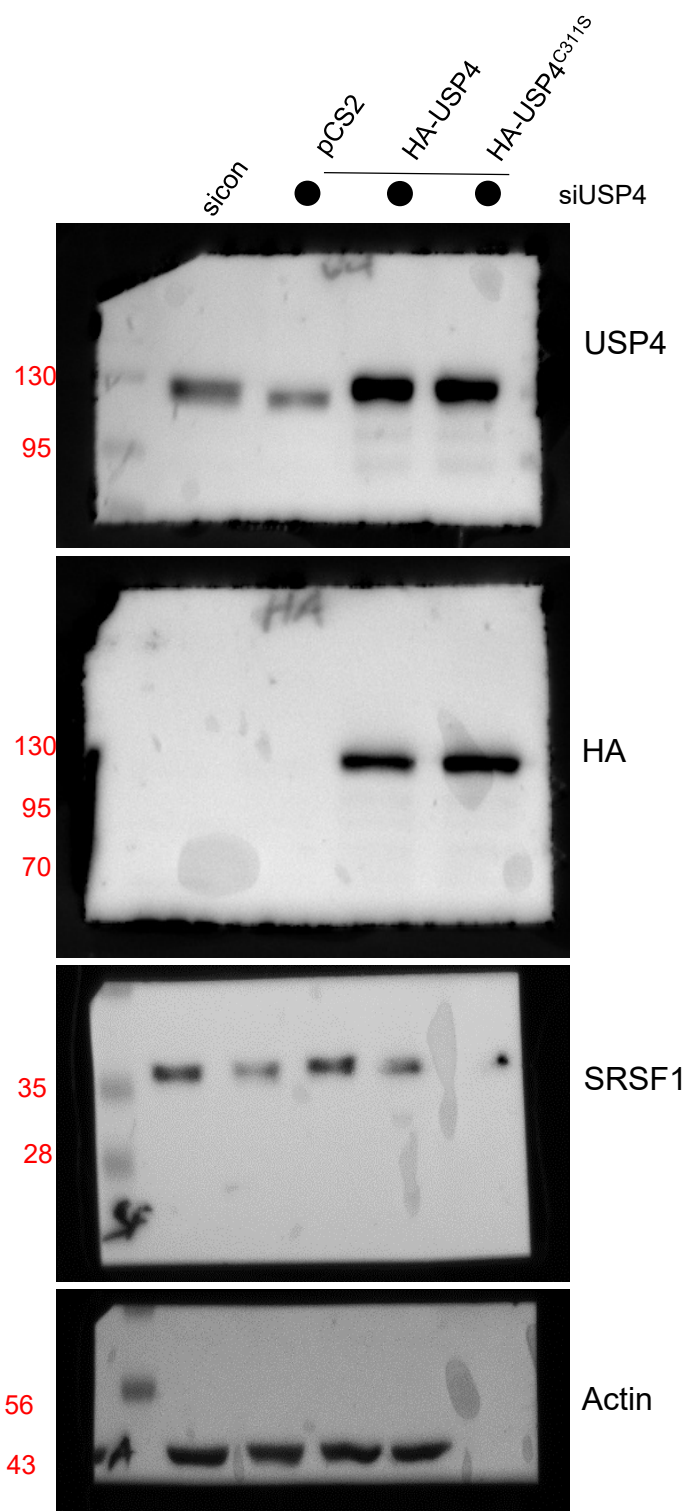

**A**

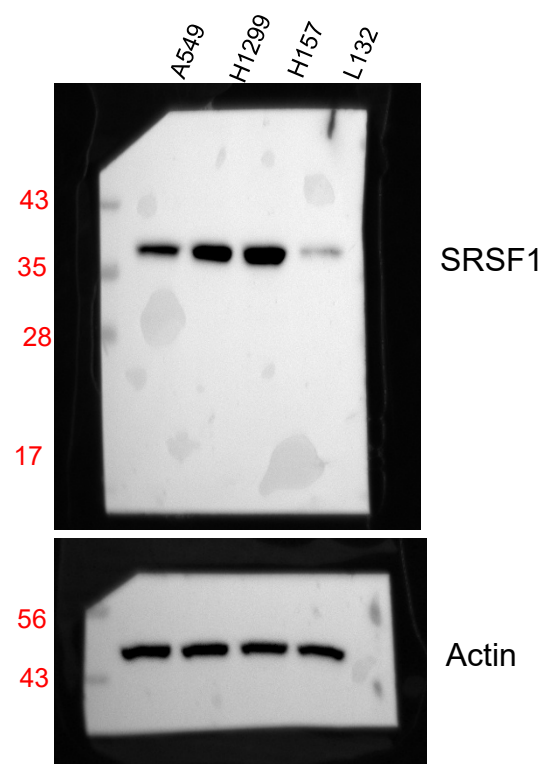

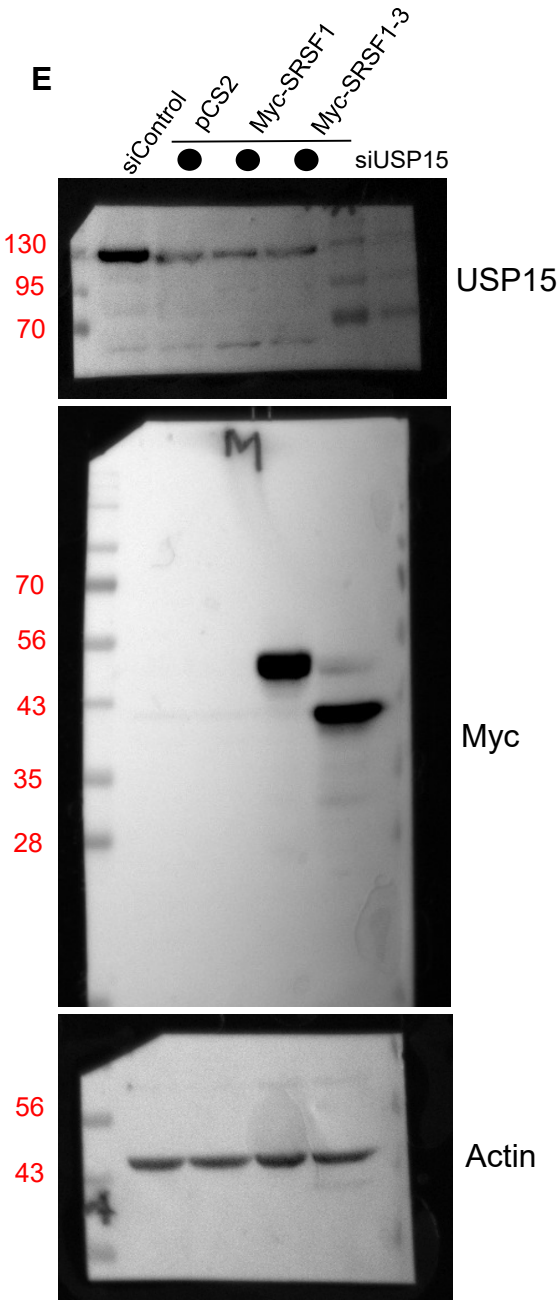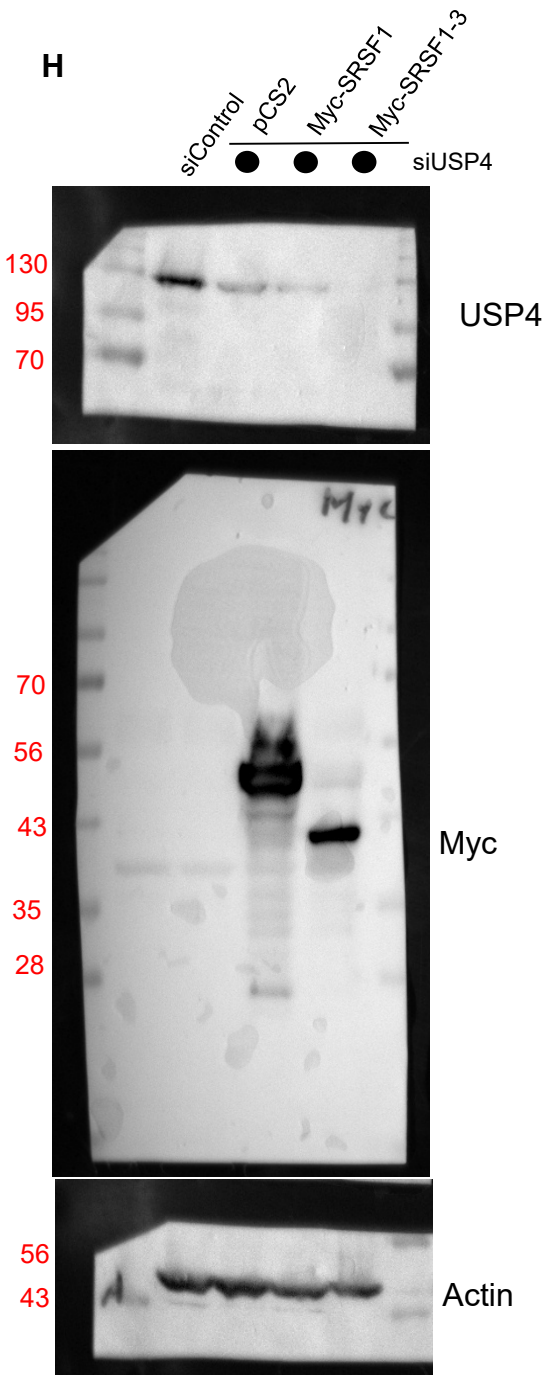

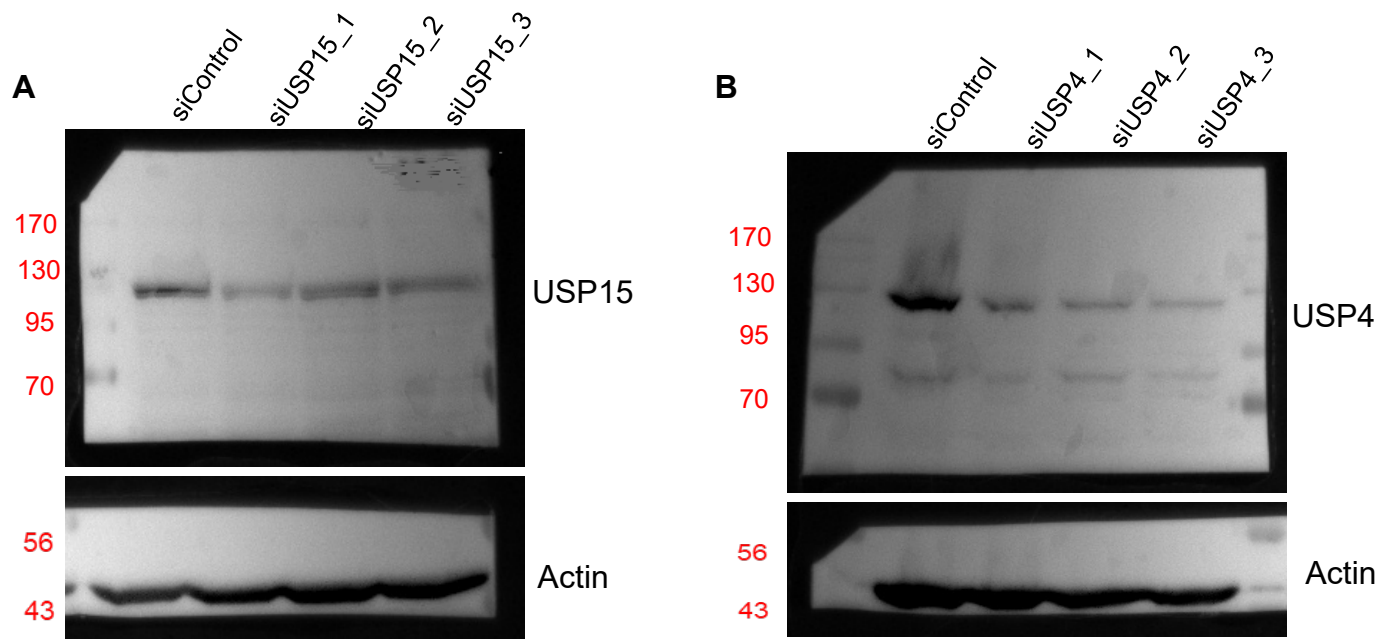

Das et al., Supplementary Figure 2.

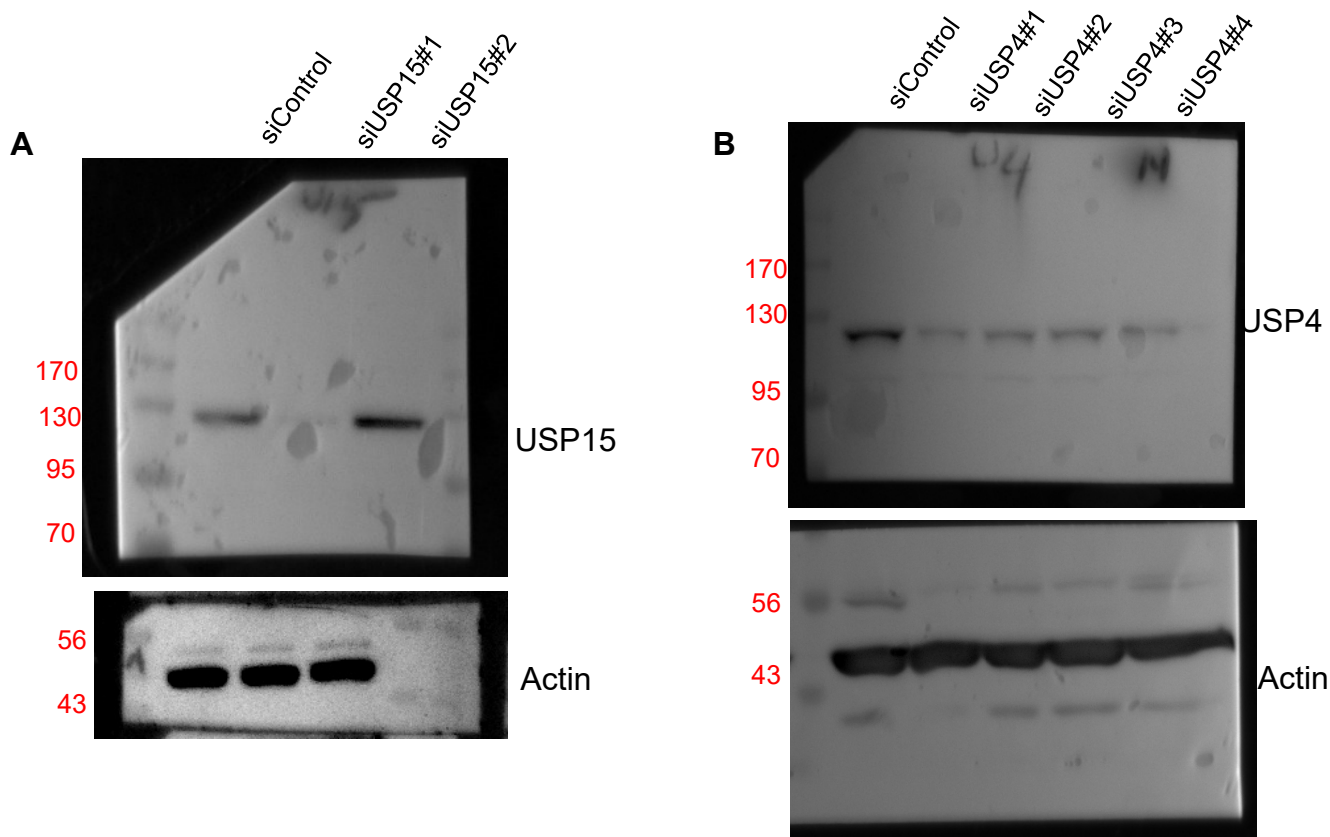

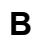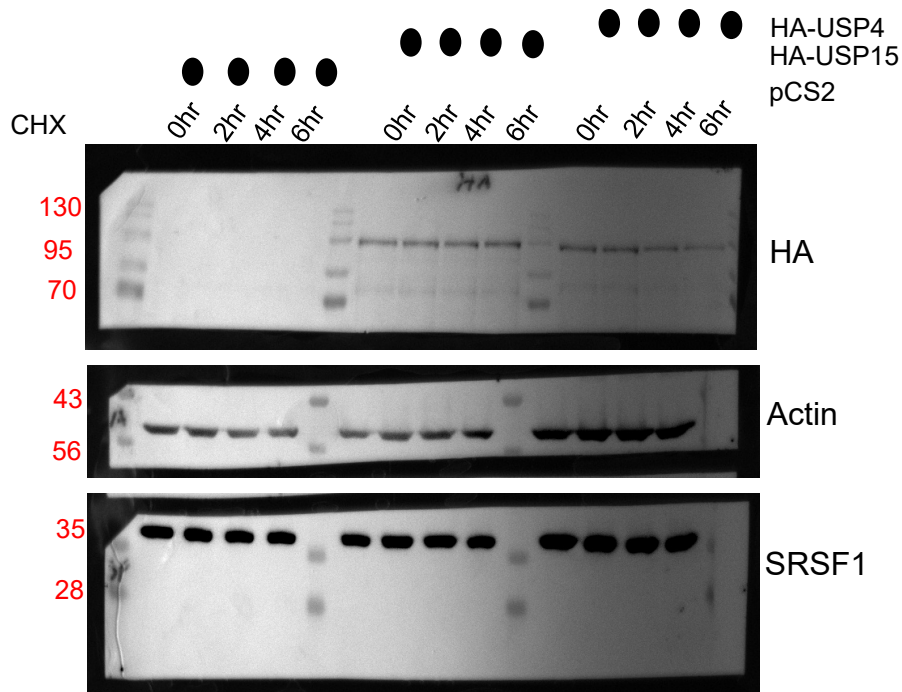

A

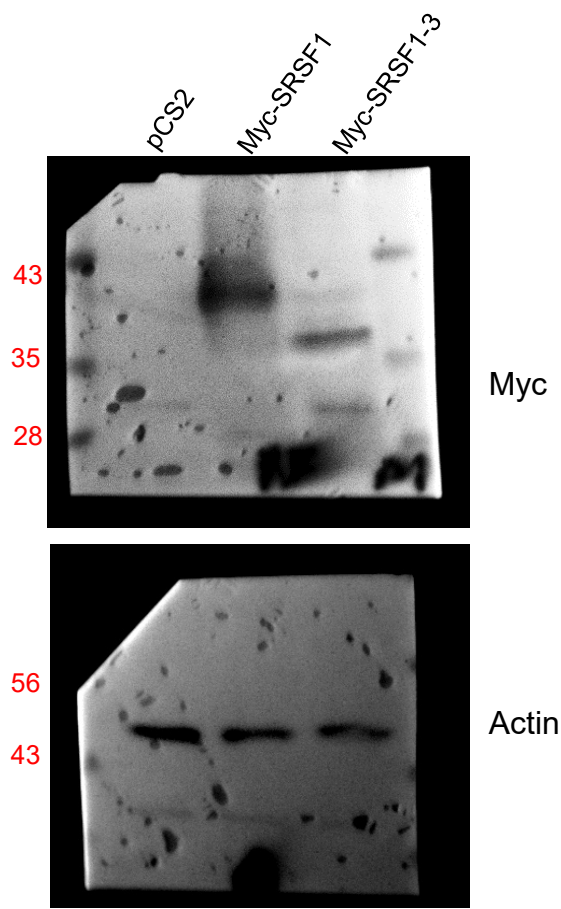

Supplement: Supplementary file 6 — uncropped original data [file 41420_2022_820_MOESM6_ESM.pdf]
