# Supplementary material for: USP15 and USP4 facilitate lung cancer cell proliferation by regulating the alternative splicing of SRSF1
Source: Cell Death Discov. 2022 Jan 13;8:24. doi: 10.1038/s41420-022-00820-0 (PMC8758713; doi:10.1038/s41420-022-00820-0)
Supplement: Supplementary file 7 — Supplementary Figure Legend [file 41420_2022_820_MOESM7_ESM.docx]

**Supplementary Figure 1. USP15 and USP4 promote cell proliferation and invasion in lung cancer cell lines**

Knockdown with three different targeted siRNAs of USP15 (A) and USP4 (B) in H157 cells was confirmed by western blot. Cell proliferation was measured after transfection of H157 cells with three independent USP15 (C) and USP4 (D) siRNAs. (E) A549 cells were transfected with USP15 and USP4 siRNA and cell proliferation was measured. (F) H157 cells were transfected with indicated siRNAs in a 6-well plate and made a vertical wound when the cells reached 100% confluency using a 200 μl pipette tip. Cells were grown continuously in the incubator and take images at regular intervals until the complete healing of the wound. (G) A549 cells were transfected with USP15 and USP4 WT along with their corresponding active site mutants and the percentage of invaded cells was determined from three independent experiments shown in (H). (**P<*0.05 and ***P*<0.01, two-tailed student’s t-test).

**Supplementary Figure 2. Global changes in alternative splicing patterns by USP4 KD**

H157 cells with stably depleted USP15 (A) or USP4 (B) were generated and the knockdown efficiency of selected clones was confirmed by western blot. (C) The percentage of the five major alternative splicings types upon USP4 knockdown are shown. (D) The relative fractions of upregulated or downregulated genes by USP4 depletion are represented. (E) The diverse cellular pathways regulated by USP4 mediated alternative splicing are classified into distinct functional groups. (F) The nucleotide sequence of human SRSF1 mRNA showing four exon structures in shaded green and the retained intron part is highlighted in red.

**Supplementary Figure 3. SRSF1 is not deubiquitinated by USP15 or USP4**

(A, B) Myc-SRSF1 and HA-USP15/HA-USP4 wild type or the active site mutants were co-expressed with His-ubiquitin and covalently modified proteins were purified on Ni-NTA-agarose under denaturing conditions. Ubiquitinated SRSF1 was detected by the anti-Myc antibody. (C) H157 cells were transfected with HA-USP15 or HA-USP4 and then treated with 100 μg/mL cycloheximide and harvested at the times indicated. Protein expressions were detected by western blot using anti-SRSF1, HA and Actin antibody.

**Supplementary Figure 4. SRSF1, but not SRSF1-3, increases the invasion of A549 cell**

(A) H157 cells were overexpressed with SRSF1 and SRSF1-3 plasmids and protein expression were confirmed by western blot. (B) A549 cells were transfected with SRSF1 and SRSF1-3 plasmids. After 24 h of transfection, cells were harvested and reseeded on the transwell. Invaded cells were visualized and imaged under a light microscope.

**Supplementary Figure 5. SRSF1, but not SRSF1-3, rescues the invasion in the USP15 or USP4 depleted A549 cell**

A549 cells were transfected with USP15 (A) or USP4 (B) siRNAs, rescued with either SRSF1 or SRSF1-3 plasmids and cell invasion was detected.
